# Can vitamin D supplementation be dangerous? 25-Hydroxyvitamin D and 1,25-hydroxyvitamin D concentration in healthy population and primary hyperparathyroidism: a single-center experience and literature review

**DOI:** 10.3389/fendo.2025.1700345

**Published:** 2025-12-04

**Authors:** Łukasz Obołończyk, Monika Berendt-Obołończyk, Krzysztof Sworczak, Izabela Karwacka-Bujak, Ewa Majerowska, Marcin Renke

**Affiliations:** 1 Department of Occupational, Metabolic Internal Diseases, Medical University of Gdańsk, Gdańsk, Poland; 2 Department of Endocrinology and Internal Medicine, Medical University of Gdańsk, Gdańsk, Poland; 3 Department of Corporate Finance, Faculty of Management, University of Gdansk, Gdańsk, Poland

**Keywords:** primary hyper parathyroidism, vitamin D, parathyroid hormone, 25 hydroxy vitamin D, 1,25 hydroxyvitamin D

## Abstract

Primary hyperparathyroidism (PHPT) is an excessive secretion of PTH caused by a defect in parathyroid cells, insensitive to the suppressive effects of hypercalcemia. The effect of 25-hydroxyvitamin D3 (25-OH-D) and skeletal and extra-skeletal conditions is well known, but the impact of 25-OH-D and 1,25-OH-D in the pathogenesis of primary hyperparathyroidism (PHPT) is still poorly researched. The study group (SG) consists of 85 patients with diagnosed PHPT. The control group (CG) consists of 51 patients without calcium-phosphate imbalance. The SG was divided into two subgroups: SG1 (patients with PHPT qualified for conservative management) and SG2 (patients who are qualified for surgical treatment). Serum PTH, calcium and phosphates concentration were assessed as well as the concentration of 25-OH-D and 1,25-OH-D. The ratio of 1,25-OH-D to 25-OH-D was introduced: vitamin D activation ratio (VD-AR). The median concentration of 25-OH-D3: 30,00 ng/mL in CG, 32,75 ng/ml in SG1 and 27,6 ng/mL in SG2. The highest 1,25-OH-D3 median concentration was found among patients in SG2 (91,1 pg/mL) lower in SG1 (64,05 pg/mL) and the lowest in CG (46,20 pg/mL). VD-AR was highest in SG2 as well, with median 3,32. Results given prove the statistically significant differences between the medians of 1,25-OH-D and VD-AR between pairs of all groups (e.g. SG1 *vs*. SG2; SG1 *vs*. CG etc.). In the logit model for calcium, the structural parameter for the VD-AR was statistically significant-the higher the value of VD-AR the greater the probability of hypercalcemia above 11 mg/dL (74.1%). In the logit model for indication for parathyroidectomy, the structural parameter for the VD-AR and iPTH was statistically significant at 0.05 significance level, which means that higher value of VD-AR the greater probability of surgery and independently the higher serum concentration of iPTH the greater probability of surgery as well (72.9%) To conclude, 1,25-OH-D serum concentration and VD-AR are significantly higher in PHPT patients than in the healthy population. 1,25-OH-D serum concentration and VD-AR are significantly higher in PHPT patients qualified to surgery than qualified to conservative treatment. Moreover, higher value of VD-AR makes greater probability of hypercalcemia above 11 mg/dL. We suggest VD-AR cut-off 3.3 as clinically relevant.

## 1 Introduction

Vitamin D (VD) has been a hot topic in the medical community. Many clinical studies have shown an association between the serum concentration of 25-hydroxyvitamin D (25-OH-D) and skeletal and extra-skeletal conditions. A positive effect of VD supplementation in malignancies, infections, and metabolic and cardiovascular diseases has been suggested; however, the causal effect is unclear (1, 2). These associations had an evident impact on two phenomena. The first is the widespread, but often uncontrolled, intake of VD preparations (3, 4). The second is the frequent assessment of serum 25-OH-D concentration (5). The Endocrine Society’s latest guidelines on VD use emphasize the need for controlled VD use and the limiting laboratory testing for 25-OH-D in healthy populations (6).

VD needs two steps (hydroxylation) to become active. The first is hydroxylation in the liver at position 25, which is a passive, poorly controlled process. The second is hydroxylation at position 1, which takes place in the kidneys and locally in many tissues. Circulating 1,25-hydroxyvitamin D (1,25-OH-D) is derived from the kidneys and is dependent on parathyroid hormone (PTH) concentration. It is noteworthy that the concentration of 25-OH-D is measured in nanograms per milliliter (ng/mL), while 1,25-OH-D is measured in picograms per milliliter (pg/mL), which is a thousand times lower. 1,25-OH-D physiologically stimulates 24-hydroxylase activity and fibroblast growth factor 23 (FGF23) production. Both of them prevent uncontrolled 1,25-OH-D production. 24-Hydroxylase inactivates 1,25-OH-D, and FGF23 suppresses the expression of 1-hydroxylase (7).

Primary hyperparathyroidism (PHPT) is an excessive secretion of PTH caused by a defect in parathyroid cells, insensitive to the suppressive effects of hypercalcemia. Clinical picture ranges from asymptomatic to classic (also called symptomatic) form with bone abnormalities, nephrolithiasis, and other symptoms related to hypercalcemia (8, 9). The asymptomatic one is much more common in well-developed countries. The epidemiology of PHPT still seems to be hard to estimate. Since the first epidemiological data in 1934, we know that PHPT is common (10). The detection of PHPT is clearly increasing in countries where there is wide access to laboratory tests and bone densitometry (11–13). Certainly, the incidence of PHPT is three to five times higher in postmenopausal women, but the reasons for this are unclear (14, 15). All symptomatic PHPT patients (if possible) should undergo parathyroidectomy. Asymptomatic patients with PHPT aged 50 years or older but with serum calcium levels of 1 mg/dL (0.25 mmol/L) above the upper limit of reference ranges, hypercalciuria, or skeletal or renal complications should be operated on as well (16). The remaining group of patients with PHPT may undergo conservative treatment. The symptomatic management of PHPT consists of bisphosphonates, denosumab, cinacalcet, and estrogen therapy or selective estrogen receptor modulators (SERMs) in postmenopausal women. Normal dietary calcium intake and VD supplementation are recommended as well (17).

The Endocrine Society’s guidelines suggest empiric (without 25-OH-D testing) VD supplementation in the general population aged 75 years and older (6). However, postmenopausal women are the most likely to develop PHPT (14, 15). To complicate the current situation, the 2022 recommendations state that in PHPT, VD should be maintained at >30 ng/mL. No upper limit of the required concentration for 25-OH-D is given.

This leads to a number of clinically relevant questions. Is it possible to achieve 25-OH-D > 30 ng/mL during autonomous PTH production? It is problematic, especially in the older female population, where PHPT is common and 25-OH-D supplementation is recommended empirically. Would the determination of serum calcium concentration prior to initiating VD make this therapy safer?

Few studies have demonstrated the behavior and meaning of 25-OH-D and 1,25-OH-D in the pathogenesis of PHPT. The purpose of our study was to evaluate what levels of both forms of VD are expected in PHPT and whether the 1,25-OH-D to 25-OH-D conversion ratio [vitamin D activation ratio (VD-AR)] is a possible new tool in the diagnosis and treatment of PHPT. Consequently, the primary research hypothesis assumes that there are significant differences in medians of examined variables (25-OH-D, 1,25-OH-D, and VD-AR) among the study and control groups. The secondary research hypothesis assumes that VD-AR influences serum calcium and the indication for parathyroidectomy.

## 2 Materials and methods

### 2.1 Study group and control group

The study was conducted from 2018 to 2023 in the Department of Endocrinology and Internal Medicine in Gdansk, Poland. The study group (SG) consisted of 85 consecutive patients diagnosed with PHPT. The control group (CG) consisted of 51 consecutive patients with non-toxic multinodular goiter, no calcium–phosphate imbalance, and no suspected parathyroid enlargement on neck ultrasound.

All patients in the CG, SG1, and SG had serum albumin, serum creatinine, and estimated glomerular filtration rate (eGFR) within the reference range. Medical records were reviewed, and no medications or diseases associated with calcium–phosphate imbalance were identified. Medical data also included the dose of vitamin D taken by patients during the last month. The mean doses did not differ statistically between the groups and did not exceed 2,000 units per day in any of them.

### 2.2 Inclusion criteria

A. Confirmed PHPT:

- documented PTH-related hypercalcemia (laboratory assessment at the same time) with

- serum intact PTH (iPTH) >69 pg/mL (laboratory reference ranges 11–69 pg/mL);

- serum total calcium >10 mg/dL (laboratory reference ranges 8.9–10.0 mg/dL);

- serum albumin concentration, serum creatinine, and eGFR in reference ranges for the healthy population;

- additionally, if serum total calcium was between 10.0 and 10.99 mg/dL, at least two independent assessments on different occasions had to be documented.

B. Age above 18 years.

### 2.3 Exclusion criteria

A. Any disease other than PHPT leading to calcium and phosphate metabolism disorders, especially secondary hyperparathyroidism (e.g., chronic renal failure) or hypercalcemia (i.e., granulomatous diseases).

B. Age below 18 years.

### 2.4 Study group subtypes

The SG was divided into two subgroups. The first comprised patients with PHPT qualified for conservative management (SG1), and the second comprised patients who qualified for surgical treatment (SG2). Serum calcium concentration above 11 mg/dL and age below 50 were indications for parathyroidectomy. Bone densitometry, abdominal ultrasonography, and urinary calcium excretion were not performed during the study, but previous medical data were reviewed and taken into account for SG1 and SG2 classifications. Patients with impaired kidney function were not included in the study (see exclusion criteria), and therefore, reduced glomerular filtration rate (GFR) was not an indication for surgery. This avoided the potential negative impact of renal failure on 1,25-OH-D concentrations.

### 2.5 Laboratory tests

Serum calcium concentration was determined by spectrophotometry using test kits from Abbott Laboratories, Irving, Texas. The coefficient of variation (CV) for intra-assay precision was <1.1%. iPTH concentration was determined by an immunochemical method using the IMMULITE intact PTH kit by Siemens, Tarrytown, New York. The CV for intra-assay precision was <5.7% (for mean iPTH range 72–662 pg/mL). No CV for higher concentrations of iPTH was mentioned. High-dose hook effect was not observed up to 500,000 pg/mL.

The concentration of 25-OH-D was determined using the Alinity chemiluminescent microparticle immunoassay (CMIA), which was used to quantify this substance in human serum and plasma on the Alinity i analyzer. The LIAISON^®^ XL 1,25 Dihydroxyvitamin D test from DiaSorin, Stillwater, Minnesota was used to determine 1,25-OH-D. This is an *in vitro* chemiluminescent immunoassay (CLIA) designed to quantify 1,25-OH-D in serum and plasma collected on Ethylenediaminetetraacetic acid (EDTA) and lithium heparin.

### 2.6 Other items

For the purposes of the study, a ratio of 1,25-OH-D to 25-OH-D was introduced. It was named “vitamin D activation ratio”, and the abbreviation VD-AR was used in the text. The units in which the concentration of both forms of VD was measured were not changed. 25-OH-D was in ng/mL, and 1,25-OH-D was in pg/mL.

### 2.7 Statistical methods

The details are in the Results section.

## 3 Results

### 3.1 Study and control groups

CG is a group of healthy volunteers, comprising 51 patients. SG1 is a group of PHPT patients qualified for conservative management, comprising 36 patients. SG2 is a group of PHPT patients qualified for parathyroidectomy, comprising 49 patients (**Tables 1**, **2**).

**Table 1:** Descriptive statistics of the study group.

	CG	SG1	SG2
Participants (n)	51	36	49
Gender (number of participants; percentage)			
- Male	6 (11.8%)	2 (5.5%)	5 (10.2%)
- Female	45 (88.2%)	34 (94.5%)	44 (89.8%)
Age (years)			
- Mean ± SD	57.1 ± 14.9	58.9 ± 15.3	60.6 ± 13.2
- Median	61	60	61

CG, control group; SG, study group; SG1, SG for conservative management; SG2, SG for surgical treatment.

**Table 2:** Descriptive statistics of the study group.

	CG					SG1					SG2				
Parameter	Mean ± SD	Median	q1	q3	IQR	Mean ± SD	Median	q1	q3	IQR	Mean ± SD	Median	q1	q3	IQR
Ca (mg/dL)	9.43 ± 0.46	9.4	9.225	9.7	0.475	10.4 ± 0.32	10.35	10.2	10.6	0.4	11.1 ± 0.94	11.1	10.8	11.5	0.75
P (mg/dL)	3.25 ± 0.46	3.2	2.9	3.6	0.68	2.6 ± 0.45	2.7	2.3	3	0.7	2.3 ± 0.46	2.3	2	2.6	0.62
iPTH (pg/mL)	36.15 ± 15.8	34.8	25.2	43.1	17.9	101.8 ± 44.9	90.8	69.5	136.25	66.7	213.5 ± 279.9	145.5	105.3	193.3	88

CG, control group; SG, study group; SG1, SG for conservative management; SG2, SG for surgical treatment; iPTH, intact parathyroid hormone.

### 3.2 Descriptive statistics

Supplementation status was assessed in each group. No statistically significant difference was found among all groups.

### 3.3 Median of the 25-OH-D, 1,25-OH-D, and VD-AR

The **Table 3** presents the values of the median and interquartile range calculated for the CG, SG1, and SG2 groups separately and together. Initially, a significant difference between the medians of the SG could be noticed. The SG2 took into account all parameters. Smaller values of medians were noticed in the CG in comparison with the SG1 and SG2 separately and together. Such results indicate that the statistical tests proved such a thesis.

**Table 3:** Median and interquartile range (in brackets) of the 25-OH-D, 1,25-OH-D, and VD-AR.

Groups	25-OH-D (ng/mL)	1,25-OH-D (pg/mL)	VD-AR
SG1	32.75 (20.35)	64.05 (25.28)	1.96 (1.28)
SG2	27.60 (14.50)	91.10 (35.75)	3.32 (2.29)
SG1+SG2	30.60 (14.05)	77.30 (35.45)	2.58 (1.95)
CG	30.00 (19.60)	46.20 (21.40)	1.54 (0.97)
SG1+SG2+CG	30.25 (15.50)	63.75 (40.90)	2.16 (1.96)

25-OH-D, 25-hydroxyvitamin D; 1,25-OH-D, 1,25-hydroxyvitamin D; VD-AR, vitamin D activation ratio; CG, control group; SG, study group; SG1, SG for conservative management; SG2, SG for surgical treatment.

### 3.4 Primary hypothesis testing

#### 3.4.1 Hypothesis testing—general introduction

Statistical analysis consists of three steps (three tests). The first step was the Kruskal–Wallis, which can be applied in the case of a lack of normal distribution of analyzed parameters and independent groups of patients (as in our study). The result indicates that the distribution of all three parameters significantly differs from each other. This determined the second step of analysis—Dunn’s test. It was used to compare whether there are differences (in distributions and 25-OH-D, 1,25-OH-D, and VD-AR in general) among these three groups. The test revealed there were significant differences between groups, so it may be of interest to test whether these differences come from those in medians. It led to the third step of testing, the Mann–Whitney U test, which proved the statistically significant differences between the medians (details below).

#### 3.4.2 The Kruskal–Wallis test

In the first step of the analysis, the Kruskal–Wallis test was applied. This non-parametric test was used for independent samples and due to the lack of normal distribution of the variables. The null hypothesis of the test assumed that the distributions of variables were the same in all groups. The alternative one assumed that distributions differ by at least one measure of the position. The result indicates that the distribution of all three parameters significantly differs from each other. Assuming a 0.05 significance level, all p-values (except one) were below this level (**Table 4**).

**Table 4:** The Kruskal–Wallis test.

Groups	25-OH-D (ng/mL)		1,25-OH-D (pg/mL)		VD-AR	
	Stat.	p-value	Stat.	p-value	Stat.	p-value
Kruskal–Wallis test						
SG1 *vs*. SG2 *vs*. CG	**6.58**	**0.04**	**60.19**	**<0.01**	**43.37**	**<0.01**
SG1+SG2 *vs*. CG	0.05	0.81	**49.46**	**<0.01**	**25.85**	**<0.01**

Stat., statistics of the test; 25-OH-D, 25-hydroxyvitamin D; 1,25-OH-D, 1,25-hydroxyvitamin D; VD-AR, vitamin D activation ratio; CG, control group; SG, study group; SG1, SG for conservative management; SG2, SG for surgical treatment.

Statistically significant differences were bolded.

#### 3.4.3 Dunn’s test

As a consequence of the different distributions among all groups, Dunn’s multiple comparison test was applied. The null hypothesis of the test assumed that there was no difference between the analyzed groups. The alternative hypothesis stated that there were significant differences between groups. Statistically significant differences among all groups in 1,25-OH-D and VD-AR were found. The analyses of 25-OH-D revealed statistically significant differences between SG1 *vs*. SG2 and SG1 *vs*. CG (**Table 5**).

**Table 5:** Dunn’s multiple comparison test.

Groups	25-OH-D (ng/mL)		1,25-OH-D (pg/mL)		VD-AR	
	Stat.	p-value	Stat.	p-value	Stat.	p-value
Dunn’s test						
SG1 *vs*. SG2	**2.55**	**0.01**	**−3.28**	**<0.01**	**−4.19**	**<0.01**
SG1 *vs*. CG	**1.67**	**0.05**	**3.82**	**<0.01**	**1.70**	**0.04**
SG2 *vs*. CG	−0.98	0.16	**7.75**	**<0.01**	**6.45**	**<0.01**
SG1+SG2 *vs*. CG	−0.23	0.41	**−7.03**	**<0.01**	**−5.08**	**<0.01**

Stat., statistics of the test; 25-OH-D, 25-hydroxyvitamin D; 1,25-OH-D, 1,25-hydroxyvitamin D; VD-AR, vitamin D activation ratio; CG, control group; SG, study group; SG1, SG for conservative management; SG2, SG for surgical treatment.

Statistically significant differences were bolded.

#### 3.4.4 The Mann–Whitney U test (Wilcoxon rank-sum test)

Previous tests revealed there were significant differences between groups, so it was important to assess whether these differences came from the differences in medians. In other words, we tested whether the differences in the medians could be a reason for the differences between groups.

The null hypothesis of the Mann–Whitney U test assumed that there were no statistically significant differences between the medians of two groups. The alternative hypothesis stated that there were significant differences between the medians of the two groups.

The results given in **Table 6** prove the statistically significant differences between the medians of 1,25-OH-D and VD-AR between pairs of all groups (e.g., SG1 *vs*. SG2 and SG1 *vs*. CG). They strongly suggest that PHPT changes serum levels of 1,25-OH-D and VD-AR. It is noteworthy that the median of 25-OH-D statistically differed between the SG1 and SG2. In the remaining cases, the null hypothesis could be rejected. The median value of 1,25-OH-D and VD-AR was the highest in the SG2, lower in the SG1, and the lowest in the CG (as in **Table 2**).

**Table 6:** The Mann–Whitney U test.

Groups	25-OH-D (ng/mL)		1,25-OH-D (pg/mL)		VD-AR	
	Stat.	p-value	Stat.	p-value	Stat.	p-value
Mann–Whitney test						
SG1 *vs*. SG2	**2.52**	**0.01**	**−3.95**	**<0.01**	**−4.48**	**<0.01**
SG1 *vs*. CG	1.69	0.09	**4.47**	**<0.01**	**2.00**	**0.05**
SG2 *vs*. CG	−1.00	0.31	**7.21**	**<0.01**	**6.20**	**<0.01**
SG1+SG2 *vs*. CG	−0.23	0.81	**−7.03**	**<0.01**	**−5.08**	**<0.01**

Stat., statistics of the test; 25-OH-D, 25-hydroxyvitamin D; 1,25-OH-D, 1,25-hydroxyvitamin D; VD-AR, vitamin D activation ratio; CG, control group; SG, study group; SG1, SG for conservative management; SG2, SG for surgical treatment.

Statistically significant differences were bolded.

### 3.5 Secondary hypothesis testing

#### 3.5.1 Secondary hypothesis testing: general information

The logit model allowed us to test whether VD-AR statistically influences marked hypercalcemia or the decision of surgery.

#### 3.5.2 The logit model

This part of the analysis is based on the logit model. The logistic model (or logit model) is an econometric model that models the log odds of an event as a linear combination of independent variables. The dependent variable of the model is a dummy variable that can have one or zero values. The independent variables (regressors) are those that can potentially influence the dependent one. The model is estimated using the maximum-likelihood method.

#### 3.5.3 The logit model for calcium

The dependent variable (serum calcium concentration) is a dummy variable equal to zero if serum concentration ranges from 10.00 to 10.99 mg/dL and one if serum concentration is 11 mg/dL or above. The structural parameter for the VD-AR was statistically significant at a 0.05 significance level because the z statistic given in a table for this parameter was greater than the critical value from the standard normal distribution (for p < 0.05, it was z > 1.96). It means that the higher the value of VD-AR, the greater the probability of hypercalcemia above 11 mg/dL. This model in 74.1% correctly predicts such a dependency (**Table 7**).

**Table 7:** The logit model for calcium.

Logit model for SG1+SG2				
Dependent variable: calcium				
Variable	Coefficient	Std. error	Z	Slope
Constant	−1.74	0.51	−3.44	–
VD-AR	0.40	0.15	2.75	0.10

VD-AR, vitamin D activation ratio.

#### 3.5.4 The logit model for indication for parathyroidectomy

The dependent variable (indication for parathyroidectomy) is a dummy variable equal to zero if conservative management and one if surgery is recommended. The structural parameters for the VD-AR (and additionally iPTH) were statistically significant at a 0.05 significance level (z in both cases greater than the critical value 1.96).

The structural parameter for the VD-AR and iPTH was statistically significant at a 0.05 significance level. It means that the higher the value of VD-AR, the greater the probability of surgery; independently, the higher the serum concentration of iPTH, the greater the probability of surgery as well. This model in 72.9% correctly predicts such a dependency (**Table 8**).

**Table 8:** The logit model for indication of surgery.

Logit model for parathyroid surgery indication				
Dependent variable: surgery				
Variable	Coefficient	Std. error	Z	Slope
Constant	−3.32	0.88	−3.79	–
iPTH	0.01	0.01	2.44	0.00

iPTH, intact parathyroid hormone.

## 4 Discussion

### 4.1 VD for the prevention of diseases

Dietary reference intakes (DRIs) are a set of various nutrient reference values (not only VD) used in the field of nutrition and health (18). Tanaka et al. used five more indices employed for DRIs in the Japanese population:

− for avoiding VD deficiency: estimated average requirement (EAR), recommended dietary allowance (RDA), and adequate intake (AI);
− for avoiding VD toxicity: tolerable upper intake level (UL); and
− for preventing lifestyle-related disease: tentative dietary goal (DG) (19).

DRIs for VD use 25-OH-D concentrations to define the abovementioned parameters (20). There are even some online tools to establish daily nutrient recommendations based on the DRIs: https://www.nal.usda.gov/human-nutrition-and-food-safety/dri-calculator. It is relevant to note that the 2024 guidelines do not recommend routine 25-OH-D testing (6). Therefore, in general, recommendations for VD supplementation avoid 25-OH-D assessment, but DRI reference values rely on it. This only confirms how complicated this issue is.

The 2024 Endocrine Society’s Clinical Practice Guidelines on VD recommends (in the general healthy population) empiric VD therapy in four groups of people: children from age 1 to 18, pregnant women, pre-diabetes patients, and people above 75 years old. In the same year, this was supported by Shah et al. in a systematic review (21). However, in December 2024, Holick wrote that these guidelines do not apply to individuals with abnormalities in calcium, phosphate, VD, and bone metabolism and that circulating concentrations of 25-OH-D should be at least 30 ng/mL with a preferred range of 40–60 ng/mL to obtain maximum extraskeletal benefits (31). Similar conclusions to the 2024 guidelines are included in our Polish Guidelines (22). It is important to underline that the recommendation against empiric VD supplementation does not mean against supplementation at all—it means against empiric supplementation above DRIs.

### 4.2 Safety concerns of VD supplementation

VD supplementation is really safe under two conditions. First, its dose must be in accordance with the recommendations, and second, there are no diseases that strongly affect its metabolism.

In the literature, there are various case reports of VD intoxications. De Vincentis et al. reported using a cumulative dose of VD 78,000,000 IU in 20 months in patients as an alternative treatment of multiple sclerosis (4). Bell et al. in 2013 described a case with consumption of 600,000 IU of VD daily for more than 3 years. It was an effect of unintentional error with 7.5 mg of cholecalciferol (300,000 IU) prescribed instead of 7.5 μg (300 IU) (23). Heister et al. described two cases of VD (cumulative dose approximately 2,500,000 and 10,000,000 IU) intoxication treated with therapeutic plasma exchange and hemodialysis (24). Of course, such examples are rare. It is noteworthy that long-term (above 6 months) VD supplementation in doses of 3,200–4,000 IU may lead to hypercalcemia in small groups of patients with a frequency of hypercalcemia of four cases per 1,000 individuals (25). Another case report of VD-related severe hypercalcemia (calcium 16.9 mg/dL, 25-OH-D > 150 ng/mL, and 1,25-OH-D > 200 pg/mL) with hypertriglyceridemia and acute pancreatitis in a 2-month-old baby was described by Rehman et al. The baby received a tablespoon of VD drops (every drop contains 400 IU of VD) instead of the recommended 400 IU (26).

In granulomatous diseases such as sarcoidosis and tuberculosis, ectopic 1-hydroxylase activity is observed in macrophages, leading to increased 1,25-OH-D production and hypercalcemia. In malignancies, the same mechanism is observed almost exclusively in lymphomas. In other malignancies, the production of PTH-related protein (PTH-rp) is a well-known cause of hypercalcemia, but the mechanism of action of PTH-rp does not involve VD activation (27).

The treatment of VD intoxication is difficult. The therapeutic purposes are as follows: reduce hypercalcemia, avoid hypovolemia, and reduce the levels of the most potent form of VD, i.e., 1,25-OH-D. The most important is discontinuation of VD and calcium supplementation, as well as proper hydration with isotonic saline and loop diuretics (after volume repletion) and use of glucocorticoids. Treatment with denosumab, bisphosphates, or calcitonin may help restore normocalcemia, but osteoclast activation is not an important mechanism of VD-related hypercalcemia. Second-line treatment with drugs that modify the activity of liver microsomal enzymes (i.e., phenobarbital, ketoconazole, chloroquine, hydroxychloroquine, and rifampicin) is rarely used (28).

Data on the incidence of VD poisoning are limited, but Sulaiman et al. detected hypercalcemia in a group of 9,902 patients from Asia and India in 150 cases. In this group, VD poisoning was found in 12 patients (8.7%) (29).

### 4.3 VD and PHPT

#### 4.3.1 25-OH-D in PHPT

Consequently, the elevated serum PTH concentrations (as in PHPT) will increase the conversion of 25-OH-D to 1,25-OH-D, so 25-OH-D may be lower than that in a healthy population. It may be considered questionable to use the term VD in the PHPT group solely based on the 25-OH-D concentration. **Table 9** contains the most important studies in 2020–2025 on the topic of VD and PHPT.

**Table 9:** Clinical studies related to VD and PHPT.

Author	N	Ca_c_ (mg/dL)	PTH (pg/mL)	25-OH-D (ng/mL)	1,25-OH-D (pg/mL)	25-OH-D lower in PHPT *vs*. CG	Comments
Kabadi UM. (2020) (30)	102	11.2 ± 0.4	128 ± 12	18 ± 2	79 ± 5	Yes	
Palermo A. (2020) (31)	41	10.8 ± 0.4	139.1 ± 49.7 SD	31.1 ± 7.8 SD	Not available	No	Hypercalcemic PHPT only included in the table
Unsal IO. (2020) (32)	180	11.2 ± 0.8	131.5 (61-922)	18.1 ± 10.7	Not available	Not available	
Meng I. (2020) (33)	40	10.7 ± 0.5	134.8 ± 46.4	26.4 ± 7.7	Not available	Yes	
Hassani S. (2020) (34)	29	11.3 ± 1.22	436.91 ± 180.35	25.68 ± 7.54	75.28 ± 23.75	Not available	
Rao SD. (2020) (35)	440	11.0 ± 0.81	155 ± 196	20.4 ± 9.34	Not available	Not available	
Lin X. (2021) (36)	457	11.34 (10.82–2.42 IQR)	168.35 (115.30–370.98 IQR)	14.03 (10.12–17.74 IQR)	Not available	Not available	
Soto-Pedre E. (2023) (37)	11616	10.82 (10.1–10.82 IQR)	73.55 (38.66–124.48 IQR)	15.22 (9.62–25.24 IQR)	Not available	No	Total group of participants, not equal for every parameter
Kolcsar M. (2024) (38)	60	10 ± 1.1	150 (IQR = 127)	21 (IQR = 18)	Not available	Not available	Normocalcemic PHPT included
Misgar RA. (2024) (39)	103	12.1 ± 1.34	332.9 ± 403.6	25.7 ± 15.12	Not available	No	
Pallone SG. (2024) (40)	70	11.2 (10.6–11.7)	114.5 (78.6–84.2)	22.1 ± 6.3	85 (70.5–119.2)	No	Before VD supplementation
Obołończyk Ł. (2025)	49^1^ 36^2^	11.1 (IQR 0.75)	145.5 (IQR 88)	27.6 (14.2 IQR)	91.1 (34.5 IQR)64.05 (22.83 IQR)	NoNo	

25-OH-D lower in PHPT *vs*. CG—25-OH-D concentration statistically lower in patients with PHPT compared to the control group.

N, number of patients; Ca_c_, corrected serum calcium; VD, vitamin D; PTH, parathyroid hormone; 25-OH-D, 25-hydroxyvitamin D; 1,25-OH-D, 1,25-hydroxyvitamin D; PHPT, primary hyperparathyroidism.

^1^PHPT for surgery (SG2).

^2^PHPT for conservative treatment (SG1).

Among the abovementioned 10 clinical studies, only in one study was 25-OH-D above the recommended 30 ng/mL, with the lowest reported concentration being 14.03 ng/mL. In our study, the 25-OH-D concentration in the SG1 and SG2 was 32.75 and 27.6 ng/mL, respectively. It is difficult to explain the statistically significant difference in 25-OH-D concentrations only between these groups. We did not find a statistically significant difference in 25-OH-D concentration between the study and control groups. A potential explanation for this finding may be the difference in serum concentrations of 25-OH-D (ng/mL) and 1,25-OH-D (pg/mL); i.e., only a small amount of 25-OH-D is needed for conversion and a significant increase in 1,25-OH-D levels. This will not markedly change the concentration of 25-OH-D. Although from a mathematical point of view this theory is correct, it cannot be the only explanation for this phenomenon. For example, physiologically, parathyroid tissue, by expressing the megalin/cubilin complex, is able to uptake 25-OH-D and other D metabolites, even when bound to binding proteins. Whether this mechanism is still active in parathyroid adenoma is unknown. In addition, the role and behavior of FGF23, a strong physiological inhibitor of 1,25-OH-D kidney production, in PHPT are still unknown. This is probably why the data on 25-OH-D concentration in PHPT are so inconsistent. In conclusion, three clinical studies did not show a statistically significant difference in 25-OH-D concentration between patients with PHPT and healthy volunteers, and two showed such a relationship. Amal Dev et al. showed that PHPT patients with 25-OH-D below 20 ng/mL had statistically significantly higher levels of PTH than other groups of patients with PHPT (with 25-OH-D 20-30 and >30 ng/mL) (41). There seems to be no convincing evidence at present that 25-OH-D concentrations are helpful in the diagnosis of PHPT. However, its high concentration is not typical for PHPT and can help to rule it out.

#### 4.3.2 1,25-OH-D and VD-AR in PHPT

1,25-OH-D circulates at a very low concentration, approximately 1,000 times lower than 25-OH-D. Moreover, it has a very short plasma half-life, which causes technical difficulties in the evaluation of 1,25-OH-D and, therefore, significantly limits its use. Two main laboratory methods are in use: immunoassays and liquid chromatography–mass spectrometry (LC–MS). The second one seems to be more resistant to interference, like cross-reactivity with 25-OH-D and 24,25-OH-D. In summary, when we use 1,25-OH-D in VD-AR, the results may vary significantly, and further studies are needed to verify our observations.

Our results clearly show that the concentration of 1,25-OH-D is the highest in the SG2 (91.1 pg/mL), followed by the SG1 (64.05 pg/mL), and lowest in the CG (46.20 pg/mL). All these differences are statistically significant. Even if we combine patients from the SG2 and SG1, the 1,25-OH-D concentration is statistically higher than that in the CG. The same relationship was observed in the studies of Kabadi and Pallone (30, 40).

The use of ratios is a well-established concept in medical research. By dividing two parameters, a derived variable is obtained, representing a form of statistical transformation. Probably the most well-known is the body mass index (BMI), which is a value that results from a person’s weight and height. For example, in endocrinology, the calcium to serum phosphorus (Ca/P) ratio was analyzed. Madeo et al. determined the Ca/P ratio above 2.55 as suggestive for the identification of patients with PHPT (sensitivity and specificity >85%). In a study by Castellano et al. in a group of 515 patients with PHPT, the mean Ca/P was 4.54, and in 88.5% of the patients, it was higher than 3.3. Moreover, high Ca/P was associated with increased biochemical and clinical severity of the disease (39, 42). Of course, the problem is more complex than Ca/P, especially in the face of normocalcemic PHPT, but it remains an intriguing issue (43).

VD-AR (i.e., 1,25-OH-D in pg/mL to 25-OH-D in ng/mL) was first used by Kabadi in 2020 (30). Our results (similarly to 1,25-OH-D) showed the highest VD-AR in the SG2 (3.32), followed by the SG1 (1.98), and the lowest in the CG (1.54). All these differences are statistically significant. Even if we combine patients from the SG2 and SG1, the VD-AR is statistically higher than that in the CG. In the previously mentioned study of Kabadi, VD-AR for the entire PHPT group was 4.4. Because of a lack of similar studies, the clear clinical meaning and reference ranges for VD-AR are hard to establish. A cut-off value for VD-AR >3.3 seems to be reasonable, and it suggests potentially severe PHPT and a higher risk of VD toxicity. VD-AR > 3.3 may also help to confirm the diagnosis of PHPT in more complicated cases.

Interestingly, the logit model revealed in our study suggests two relationships. First, a higher value of VD-AR indicates a greater probability of hypercalcemia above 11 mg/dL (this model in 74.1% correctly predicts such a dependency). Second, the higher the value of VD-AR, the greater the probability of surgery, and independently, the higher the serum concentration of iPTH, the greater the probability of surgery as well (this model in 72.9% correctly predicts such a dependency). To clarify, it does not mean that VD-AR is a new indication for parathyroidectomy; rather, it should be considered that 1,25-OH-D and consequently VD-AR are strongly involved in the pathomechanism of PHPT.

#### 4.3.3 VD supplementation in PHPT

The vitamin D supplementation in PHPT remains a subject of ongoing debate and lacks a definitive consensus. The general answer is probably yes. In 2021, Song et al., in a systematic review and meta-analysis of 11 studies, showed that VD supplementation in patients with PHPT and VD deficiency (25-OH-D < 20 ng/mL) significantly reduces PTH and alkaline phosphatase levels without causing hypercalcemia and hypercalciuria (44). However, in this meta-analysis, in only one study, the mean serum calcium concentration exceeded 11.0 mg/dL. In the previously cited study by Pallone, VD supplementation in the PHPT group did not increase serum calcium level, decreased serum PTH level, and surprisingly decreased 1,25-OH-D level. This effect was hard to explain, and FGF23-mediated VD deactivation mechanisms may be involved.

The second important question is how to select PHPT patients who are at high risk of VD overdosing or toxicity. The assessment of 25-OH-D and 1,25-OH-D (with VD-AR) may help to avoid such problems. However, patients with “resistance to VD” with an all-time low 25-OH-D concentration could be candidates for PHPT screening. In this group, high VD-AR may be a reason for the constant activation of VD.

## 5 Conclusions

The main limitation of our work is that it relies on an “unstable” hormone. 1,25-OH-D has a short half-life and is present in the body in low concentrations, making it susceptible to laboratory errors. Moreover, it only takes into account the current laboratory test results without the possibility of assessing the organ complications of PHPT, which could change the classification to the SG1 or SG2. However, the research results are quite clear and allow us to draw the following conclusions.

1,25-OH-D serum concentration and VD-AR are significantly higher in PHPT patients than in the healthy population. Additionally, 1,25-OH-D serum concentration and VD-AR are significantly higher in PHPT patients qualified for surgery than those qualified for conservative treatment. Moreover, an increased VD-AR is associated with hypercalcemia above 11 mg/dL.

The evidence remains preliminary for 1,25-OH-D assessment to be considered a recommendation. On the basis of these results, we suggest assessing both types of VD in patients with PHPT because the higher 1,25-OH-D and VD-AR may be related to more severe PHPT. We suggest the VD-AR cut-off of 3.3 as clinically relevant.

## Data Availability

The raw data supporting the conclusions of this article will be made available by the authors, without undue reservation.
